# Polymorphisms in Toll-like receptor genes are associated with vitiligo

**DOI:** 10.3389/fgene.2015.00278

**Published:** 2015-09-09

**Authors:** Tanel Traks, Maris Keermann, Maire Karelson, Ranno Rätsep, Ene Reimann, Helgi Silm, Eero Vasar, Sulev Kõks, Külli Kingo

**Affiliations:** ^1^Department of Dermatology, University of TartuTartu, Estonia; ^2^Centre of Excellence for Translational Medicine, University of TartuTartu, Estonia; ^3^Clinic of Dermatology, Tartu University HospitalTartu, Estonia; ^4^Department of Physiology, University of TartuTartu, Estonia; ^5^Department of Pathophysiology, University of TartuTartu, Estonia; ^6^Department of Reproductive Biology, Estonian University of Life SciencesTartu, Estonia

**Keywords:** vitiligo, autoimmune, Toll-like receptor, SNP, genetic association study

## Abstract

**Background:** The members of Toll-like receptor (TLR) family are responsible for recognizing various molecular patterns associated with pathogens. Their expression is not confined to immune cells and have been detected in skin cells such as keratinocytes and melanocytes. As part of a generated response to pathogens, TLRs are involved in inducing inflammatory mediators to combat these threats. It is therefore not surprising that TLRs have been implicated in inflammatory skin diseases, including atopic dermatitis and psoriasis. Likewise, as key players in autoimmunity, they have been associated with a number of autoimmune diseases. Based on this, the role of TLRs in vitiligo could be suspected, but is yet to be clearly established.

**Methods:** In order to conduct a genetic association analysis, 30 SNPs were selected from TLR1-TLR8 and TLR10 regions to be genotyped in Estonian case-control cohort consisting of 139 vitiligo patients and 307 healthy control individuals. The patients were further analyzed in subgroups based on sex, age of onset, occurrence of vitiligo among relatives, extent of depigmented areas, vitiligo progression activity, appearance of Köbner's phenomenon, existence of halo naevi, and incidence of spontaneous repigmentation.

**Results:** The most notable finding came with SNP rs179020 situated in TLR7 gene, that was associated in entire vitiligo (Padj = 0.0065) and also several subgroup analyses. Other single marker and haplotype analyses pointed to TLR3, TLR4, and TLR10 genes.

**Conclusions:** This study investigated the genetic regions of nine TLR genes in relation to vitiligo susceptibility. The main results were the associations of TLR7 SNPs with vitiligo, while several other associations were obtained from the remaining TLR gene regions. This suggests that in addition to other inflammatory skin diseases, TLRs affect the development of vitiligo, thus making them interesting targets for future research.

## Introduction

Vitiligo is a chronic disease manifested by distinctive lightening of the skin and the cause to these depigmented areas is in the loss of melanocytes. It is considered to be a complex disease, as there is a multitude of genetic and environmental factors involved in this process (Boissy and Spritz, 2009). The precise set of contributors is yet to be determined, but the general consensus approaches it as an autoimmune disease (Le Poole and Luiten, 2008). This notion is supported by the findings that vitligo susceptibilty genes primarily include immune-related genes and by the frequent co-occurrence with other autoimmune diseases (Spritz, 2011). It also implies that a shared genetic background with different self-reactive pathologies can be expected. At the same time, the importance of environmental risk factors remains, in that the concordance in monozygotic twins is only 23% (Alkhateeb et al., 2003).

Toll-like receptors (TLRs) function as critical components of innate immune system by recognizing various molecular motifs associated with pathogens or tissue injury (Takeda et al., 2003). This leads to the induction of inflammatory and antimicrobial innate immune responses as well as antigen-specific adaptive immune mechanisms (Akira and Takeda, 2004). Crucial to this system is the discrimination between self and foreign antigens and regulatory control over downstream immune activation. The breakdown of these processes causes autoimmunity and TLRs are suggested to have a pivotal function in this pathology (Pradhan et al., 2012; Liu et al., 2014). Indeed, they have been associated with various autoimmune diseases and vitiligo among them (Kang et al., 2009; Yu et al., 2009). Specifically, TLR3 that is expressed in melanocytes may sense viral infection and induce apoptosis along with local immune response, that contributes to vitiligo development (Yu et al., 2009). TLR4 in melanocytes could react to endogenous heat-shock proteins and initiate autoimmunity (Yu et al., 2009). Activation of TLR7 may trigger melanocyte apoptosis and thus the appearance of vitiligo symptoms (Kang et al., 2009).

The expression of TLRs has been detected in keratinocytes, melanocytes, and Langerhans cells of the skin (Hari et al., 2010). As could be expected, they have been implicated in a several inflammatory skin diseases such as atopic dermatitis, psoriasis, and acne vulgaris (Miller, 2008; Hari et al., 2010). On a genetic level, only a few studies have been conducted, yielding support for TLR2 involvement in atopic dermatitis (Oh et al., 2009) and TLR9 in psoriasis treatment response (Romaní et al., 2015). Still, a recent report has found *TLR2* and *TLR4* single-nucleotide polymorphisms (SNPs) to be associated with vitiligo (Karaca et al., 2013).

Considering this background, the aim of this study was to explore possible genetic associations between TLR gene polymorphisms and vitiligo. Thirty SNPs from six genetic loci containing *TLR1-TLR8* and *TLR10* genes were selected for genotyping and subsequent association analyses.

## Materials and methods

### Study sample

To assemble the study sample, 139 vitiligo patients and 307 healthy control individuals were enrolled at the Department of Dermatology, University of Tartu, Estonia. All subjects were unrelated, of Caucasian origin, and living in Estonia. Vitiligo diagnosis was based on characteristic skin depigmentation at typical locations and whiteness of skin lesions under Wood's lamp. To conduct additional analyses, the patients were separated into subgroups according to several characteristics. Female (*n* = 94) and male (*n* = 45) patients were analyzed independently against their respective controls (*n* = 168; *n* = 139). Early onset vitiligo (*n* = 41) was assigned in case the symptoms appeared before the age of 20 and late onset (*n* = 98) in case 20 or after. Familial vitiligo (*n* = 36) was determined by occurrence of vitiligo in patients' relatives and the absence of vitiligo among them indicated the sporadic cases (*n* = 102). One patient fell in neither category, since family data was not available. The extent of affected areas was the basis of next two groups: extent < 10% (*n* = 71) and extent ≥ 10% (*n* = 68). The patients were classified to have active vitiligo (*n* = 96) in case new areas of depigmentation had appeared during the previous 3 months and stable vitiligo (*n* = 43) if new areas or enlargement of previously existing depigmentation had not occurred during this period. Patients with Köbner's phenomenon, manifested by development of new vitiligo patches at sites of skin injury, comprised the Köbner positive group (*n* = 23). The occurrence of halo naevi (*n* = 19) and spontaneous repigmentation (*n* = 38) were the last distinguishing factors. The control group was recruited at University of Tartu from medical students, health care personnel and patients presenting at the dermatological outpatient clinic with mild expression of either facial teleangiectasis or skin tags.

The Human Research Ethics Committee of the University of Tartu approved the study and informed consent was obtained from all participants.

### SNP selection and genotyping

SNPbrowser version 3.5 was used for SNP selection and for SNPlex™ (Applied Biosystems) assay pool design. The SNPs were located in six loci that contain the genes *TLR1-TLR8* and *TLR10* (Table 1). The SNPs were selected to evenly cover each locus and non-synonymous SNPs were always preferred. Genomic DNA was extracted from 9 ml blood samples and Applied Biosystems SNPlex™ method was used for genotyping (Tobler et al., 2005).

**Table 1:** **Characteristics of studied SNPs**.

**SNP**	**Chr region**	**Base position**	**Gene**	**Minor/major alleles**	**Function**	**MAF**
rs2247421	1q41	221406312		G/T		0.349
rs4140967	1q41	221418246		G/C		0.312
rs10776482	4p14	38451180	TLR10	C/T	coding-synonymous	0.147
rs7694115	4p14	38455489	TLR10	G/A	intron	0.382
rs7660429	4p14	38458876	TLR10	G/C	intron	0.251
rs12233670	4p14	38463611		T/C		0.138
rs10005625	4p14	38471547		T/C		0.003
rs5743828	4p14	38503915	TLR6	A/G	near-gene-3 (failed)	0.185
rs5743810	4p14	38506745	TLR6	T/C	missense (Ser249Pro)	0.385
rs6531668	4p14	38509990		A/G		0.428
rs6531670	4p14	38511417		C/T		0.441
rs1992253	4q31.3	154822378		T/C		0.031
rs4585282	4q31.3	154832096	TLR2	T/C	intron	0.01
rs7694512	4q31.3	154837310	TLR2	T/G	intron	0.362
rs13123230	4q31.3	154840035	TLR2	G/A	intron	0.388
rs1339	4q31.3	154851013	RNF175	G/A	missense (Met195Ile)	0.261
rs2289318	4q31.3	154853184	RNF175	C/G	intron	0.255
rs11721827	4q35.1	187228131	TLR3	C/A	intron	0.198
rs6552950	4q35.1	187231850	TLR3	G/A	intron	0.253
rs4608848	4q35.1	187247098		C/T		0.383
rs1519309	4q35.1	187252083		G/A		0.298
rs10759932	9q33.1	119504965	TLR4	C/T	near-gene-5	0.196
rs5030728	9q33.1	119514103	TLR4	A/G	intron	0.269
rs5935436	Xp22.2	12793812	TLR7	T/C	near-gene-5	0.069
rs179020	Xp22.2	12799778	TLR7	T/C	intron	0.26
rs179013	Xp22.2	12811392	TLR7	T/C	intron	0.266
rs179008	Xp22.2	12813580	TLR7	T/A	missense (Leu11Gln)	0.279
rs10127190	Xp22.2	12816916	TLR7	A/T	UTR-3	0.004
rs850632	Xp22.2	12819487		C/T		0.264
rs179003	Xp22.2	12823862		G/T		0.196

*MAF, minor allele frequency among vitiligo patients and healthy controls*.

*RNF175, ring finger protein 175*.

### Data analysis

The Haploview v4.2 program was used for Hardy-Weinberg equilibrium (HWE) calculations in control group and also for allelic association and haplotype association tests between groups of patients and controls (Barrett et al., 2005). The Solid spine of LD algorithm integrated in Haploview v4.2 was applied to define the haplotype blocks and the resulting blocks were used in the haplotype association test. Differences in allele or haplotype frequencies between cases and controls were assessed by chi square test. The statistical significance threshold was set to 0.05 for all tests. Ten thousand permutations were performed to correct *p*-values for errors of multiple testing. SNPs of each of the studied regions were analyzed separately.

## Results

The genotyping procedure provided data for 30 SNPs and 29 of them qualified for subsequent statistical analysis. A single SNP rs5743828 was excluded due to deviation from Hardy-Weinberg equilibrium. All the remaining markers met the inclusion criteria for minor allele frequency (MAF > 1%) and Hardy-Weinberg equilibrium (Hardy-Weinberg *p* > 0.01).

### Allelic association analysis

Single marker associations were present in all studied regions except for chromosome 1q41. Four associations were revealed when analysing the entire vitiligo group and the rest were produced when analysing by different subgroups. The results obtained from entire vitiligo, early onset, late onset, familial, sporadic, extent < 10%, extent ≥ 10%, active, and stable vitiligo analyses are presented in Table 2.

**Table 2:** **Results of allelic association analysis**.

**SNP**	**Gene**	**Control MAF**	**Vitiligo**		**Early onset**		**Late onset**		**Familial**		**Sporadic**		**Extent** < **10%**		**Extent** ≥ **10%**		**Active**		**Stable**	
			**MAF**	***P*-value**	**MAF**	***P*-value**	**MAF**	***P*-value**	**MAF**	***P*-value**	**MAF**	***P*-value**	**MAF**	**P-value**	**MAF**	***P*-value**	**MAF**	***P*-value**	**MAF**	***P*-value**
rs2247421		0.354	0.338	0.6434	0.329	0.6583	0.342	0.7544	0.319	0.56	0.343	0.7765	0.359	0.9097	0.316	0.4009	0.344	0.7934	0.326	0.6039
rs4140967		0.317	0.300	0.6069	0.288	0.5876	0.305	0.7525	0.292	0.6562	0.301	0.6666	0.309	0.8452	0.291	0.5508	0.298	0.6131	0.305	0.8184
rs10776482	TLR10	0.135	0.174	0.1312	0.141	0.8811	0.188	0.0727	0.206	0.1116	0.165	0.2962	0.129	0.8524	0.220	**0.0134**	0.170	0.2306	0.183	0.2399
rs7694115	TLR10	0.359	0.431	**0.0446**	0.402	0.4472	0.443	**0.0387**	0.457	0.1089	0.421	0.1193	0.464	**0.0214**	0.396	0.4323	0.405	0.2541	0.488	**0.0226**
rs7660429	TLR10	0.235	0.285	0.1147	0.275	0.4332	0.289	0.1306	0.292	0.2896	0.281	0.1994	0.353	0.0045^*^	0.216	0.641	0.263	0.4312	0.333	0.0506
rs12233670		0.131	0.153	0.3775	0.100	0.4323	0.175	0.125	0.157	0.5452	0.153	0.4235	0.138	0.8379	0.169	0.2458	0.147	0.5678	0.167	0.3725
rs10005625		0.005	0	0.244	0	0.5252	0	0.3286	0	0.5516	0	0.3188	0	0.4065	0	0.4133	0	0.3311	0	0.5202
rs5743810	TLR6	0.387	0.383	0.9257	0.476	0.1217	0.344	0.2863	0.333	0.3796	0.405	0.6421	0.370	0.7116	0.397	0.8196	0.384	0.9546	0.381	0.9218
rs6531668		0.428	0.427	0.9809	0.549	**0.0385**	0.375	0.1948	0.347	0.1897	0.460	0.4265	0.420	0.8709	0.434	0.899	0.417	0.7842	0.451	0.6885
rs6531670		0.449	0.419	0.4482	0.348	0.1174	0.451	0.9618	0.538	0.2147	0.380	0.1166	0.436	0.8035	0.400	0.3587	0.410	0.3911	0.439	0.8793
rs1992253		0.038	0.016	0.0885	0.014	0.2821	0.017	0.1609	0.016	0.3606	0.016	0.1387	0.016	0.2234	0.015	0.1974	0.017	0.1797	0.012	0.2442
rs4585282	TLR2	0.008	0.015	0.3593	0.025	0.1572	0.011	0.7506	0.015	0.565	0.015	0.3959	0.021	0.1746	0.008	0.9788	0.005	0.7068	0.036	**0.0264**
rs7694512	TLR2	0.359	0.369	0.7681	0.300	0.2988	0.399	0.3212	0.343	0.7893	0.378	0.6389	0.400	0.3641	0.336	0.6198	0.326	0.4122	0.464	0.0613
rs13123230	TLR2	0.391	0.379	0.7193	0.325	0.2506	0.401	0.8125	0.357	0.577	0.385	0.8712	0.407	0.7319	0.348	0.3576	0.339	0.1945	0.465	0.1919
rs1339	RNF175	0.262	0.260	0.9675	0.230	0.5552	0.273	0.7578	0.266	0.9433	0.256	0.8729	0.262	0.9927	0.258	0.9422	0.250	0.7609	0.284	0.6815
rs2289318	RNF175	0.253	0.257	0.8998	0.317	0.2166	0.232	0.5493	0.229	0.6516	0.270	0.6439	0.239	0.7319	0.276	0.5837	0.268	0.6762	0.233	0.6782
rs11721827	TLR3	0.199	0.196	0.9199	0.171	0.5626	0.207	0.8189	0.167	0.5294	0.198	0.9713	0.246	0.2359	0.145	0.1635	0.189	0.7641	0.214	0.7625
rs6552950	TLR3	0.266	0.226	0.2106	0.244	0.678	0.218	0.1912	0.271	0.9164	0.206	0.0967	0.217	0.2419	0.234	0.4645	0.225	0.2745	0.226	0.4409
rs4608848		0.371	0.409	0.2841	0.450	0.1705	0.392	0.6016	0.472	0.0942	0.385	0.7209	0.449	0.0875	0.368	0.9431	0.400	0.4701	0.429	0.3068
rs1519309		0.306	0.281	0.4544	0.288	0.7363	0.278	0.4653	0.250	0.3275	0.295	0.7707	0.290	0.7108	0.272	0.4359	0.271	0.3639	0.302	0.946
rs10759932	TLR4	0.196	0.196	0.9954	0.229	0.5192	0.181	0.6712	0.217	0.7025	0.188	0.8277	0.173	0.5674	0.221	0.5543	0.188	0.815	0.214	0.7172
rs5030728	TLR4	0.287	0.222	0.0563	0.203	0.125	0.231	0.1586	0.129	0.0077^*^	0.256	0.4228	0.277	0.8198	0.169	0.0082^*^	0.228	0.1355	0.206	0.1556
rs5935436	TLR7	0.079	0.048	0.0977	0.061	0.5662	0.043	0.0881	0.057	0.5162	0.045	0.1103	0.074	0.8313	0.022	**0.0192**	0.048	0.1487	0.049	0.3313
rs179020	TLR7	0.225	0.361	8.5E-5^*^	0.226	0.9826	0.416	1.5E-6^*^	0.293	0.2365	0.378	8.7E-5^*^	0.366	**0.0014**	0.356	**0.004**	0.342	**0.0027**	0.406	**0.0012**
rs179013	TLR7	0.243	0.317	0.0208	0.305	0.2214	0.321	**0.0291**	0.319	0.1548	0.319	**0.0326**	0.310	0.0979	0.324	0.051	0.328	**0.019**	0.291	0.3339
rs179008	TLR7	0.258	0.326	**0.041**	0.325	0.2038	0.326	0.0705	0.309	0.3689	0.330	0.0517	0.329	0.0915	0.323	0.1408	0.341	**0.0293**	0.293	0.5055
rs10127190	TLR7	0.005	0.004	0.7874	0	0.5252	0.005	0.9723	0	0.5516	0.005	1.0	0.007	0.7518	0	0.4133	0	0.3311	0.012	0.4391
rs850632		0.264	0.264	0.9861	0.305	0.4323	0.247	0.6479	0.292	0.6149	0.248	0.6448	0.271	0.8563	0.257	0.8747	0.255	0.8104	0.286	0.6721
rs179003		0.188	0.215	0.3799	0.243	0.2601	0.202	0.6825	0.219	0.5564	0.216	0.4165	0.189	0.9961	0.242	0.1801	0.235	0.1756	0.167	0.6553

P ≤ 0.05 bolded;

* *P ≤ 0.05 after 10 000 permutations*.

The SNPs of region 4p14 provided 10 associations. SNP rs7694115 was associated in entire vitiligo group [*p* = 0.0446, odds ratio (OR) 1.35, 95% confidence interval (CI) 1.01–1.81] and also in stable (*p* = 0.0226, OR 1.7, CI 1.07–2.69), male (*p* = 0.006, OR 1.96, CI 1.21–3.17), extent < 10% (*p* = 0.0214, OR 1.55, CI 1.06–2.24), and late onset (*p* = 0.0387, OR 1.42, CI 1.02–1.97) subgroups. SNP rs10776482 was associated in extent ≥ 10% (*p* = 0.0134, OR 1.81, CI 1.13–2.9) and Köbner (*p* = 0.023, OR 2.28, CI 1.1–4.71) subgroups. SNP rs7660429 was associated in extent < 10% (*p* = 0.0045, OR 1.77, CI 1.19–2.64), rs12233670 in male (*p* = 0.0095, OR 2.28, CI 1.21–4.29), and rs6531668 in early onset (*p* = 0.0385, OR 1.63, CI 1.02–2.59) subgroups. The association of rs7660429 in extent < 10% remained significant after correcting for multiple testing (*p* = 0.0469).

In 4q31.3 region, a single SNP rs4585282 was associated in stable (*p* = 0.0264, OR 4.5, CI 1.05–19.17) and Köbner (*p* = 0.0162, OR 6.07, CI 1.14–32.27) subgroups.

A single SNP rs6552950 of 4q35.1 region was associated in male subgroup (*p* = 0.0213, OR 0.49, CI 0.27–0.91).

Next, a single SNP rs5030728 of 9q33.1 region was associated in female (*p* = 0.0384, OR 0.62, CI 0.39–0.98), familial (*p* = 0.0077, OR 0.37, CI 0.17–0.79), and extent ≥ 10% (*p* = 0.0082, OR 0.51, CI 0.3–0.84) subgroups. The last two associations remained significant after correcting for multiple testing (*p* = 0.0165 and *p* = 0.0227, respectively).

The most notable results occurred in Xp22.2 region and concerned the SNP rs179020. It was associated in entire vitiligo group (*p* = 8.5E-5, OR 1.95, CI 1.39–2.73) and also active (*p* = 0.0027, OR 1.8, CI 1.22–2.64), stable (*p* = 0.0012, OR 2.36, CI 1.39–4.03), female (*p* = 0.0483, OR 1.52, CI 1–2.31), male (*p* = 8.8E-5, OR 3.1, CI 1.73–5.55), sporadic (*p* = 8.7E-5, OR 2.1, CI 1.44–3.06), extent < 10% (*p* = 0.0014, OR 1.99, CI 1.3–3.06), extent ≥ 10% (*p* = 0.004, OR 1.91, CI 1.22–2.97), Köbner (*p* = 0.0265, OR 2.3, CI 1.08–4.9), halo (*p* = 0.0056, OR 2.69, CI 1.3–5.54), and late onset (*p* = 1.5E-6, OR 2.46, CI 1.69–3.56) subgroups. The results in entire vitiligo, male, sporadic, and late onset groups remained significant after correcting for multiple testing (*p* = 0.0065, *p* = 0.0479, *p* = 0.0078, and *p* = 0.0004, respectively). In addition, two other SNPs on this region were associated in entire vitiligo group. These were rs179013 (*p* = 0.0208, OR 1.45, CI 1.06–1.98) and rs179008 (*p* = 0.041, OR 1.39, CI 1.01–1.9). The former was also associated in active (*p* = 0.019, OR 1.52, CI 1.07–2.17), sporadic (*p* = 0.0326, OR 1.46, CI 1.03–2.07) and late onset (*p* = 0.0291, OR 1.48, CI 1.04–2.1) vitiligo subgroups and the latter in active vitiligo subgroup (*p* = 0.0293, OR 1.48, CI 1.04–2.12). Finally, rs5935436 was associated with vitiligo in females (*p* = 0.0102, OR 0.3, CI 0.11–0.79) and in extent ≥ 10% group (*p* = 0.0192, OR 0.27, CI 0.08–0.87).

### Haplotype association analysis

Haplotype blocks were formed by SNPs of all studied regions. Similarly, to single marker analysis, haplotype associations were revealed in all regions except for 1q41. Seven associations were obtained in entire vitiligo group and others occurred in different vitiligo subgroups. The composition of haplotype blocks and results of haplotype association analysis in entire vitiligo group are presented in Table 3.

**Table 3:** **Results of haplotype analysis in entire vitiligo group**.

**Chr region**	**Block**	**Haplotype**				**Freq case**	**Freq control**	***P*-value**
1q41	Block 1	rs2247421	rs4140967					
		A	C			0.662	0.644	0.6107
		C	G			0.298	0.316	0.5817
		C	C			0.040	0.038	0.8593
4p14	Block 1	rs10776482	rs7694115	rs7660429				
		A	A	C		0.547	0.628	**0.021**
		A	G	G		0.285	0.235	0.1147
		G	G	C		0.146	0.127	0.4271
		G	A	C		0.019	0.009	0.2357
	Block 2	rs12233670	rs10005625	rs5743810	rs6531668			
		C	C	G	G	0.431	0.447	0.6687
		C	C	A	A	0.376	0.382	0.8634
		T	C	G	G	0.144	0.124	0.4179
		C	C	G	A	0.040	0.040	0.9875
4q31.3	Block 1	rs1992253	rs4585282	rs7694512	rs13123230			
		C	C	G	A	0.604	0.599	0.8859
		C	C	T	G	0.346	0.322	0.4869
		C	C	G	G	0.022	0.035	0.3251
		T	C	T	G	0.013	0.034	0.0748
	Block 2	rs1339	rs2289318					
		T	G			0.494	0.490	0.9195
		C	G			0.249	0.257	0.8088
		T	C			0.251	0.249	0.9398
4q35.1	Block 1	rs11721827	rs6552950	rs4608848	rs1519309			
		A	A	T	T	0.248	0.253	0.8802
		A	G	T	T	0.195	0.233	0.2055
		C	A	C	T	0.192	0.171	0.4556
		A	A	C	C	0.143	0.166	0.3951
		A	A	T	C	0.133	0.116	0.4745
		A	G	C	T	0.026	0.013	0.1655
		A	A	C	T	0.038	0.007	7.0E-4^*^
		C	A	T	T	0.012	0.015	0.6581
9q33.1	Block 1	rs10759932	rs5030728					
		T	G			0.575	0.517	0.1331
		T	A			0.223	0.287	0.0596
		C	G			0.203	0.196	0.8298
Xp22.2	Block 1	rs5935436	rs179020					
		C	G			0.619	0.699	**0.0187**
		C	A			0.333	0.222	5.0E-4^*^
		T	G			0.039	0.076	**0.0402**
	Block 2	rs179013	rs179008					
		G	A			0.672	0.741	**0.034**
		A	T			0.313	0.242	**0.0275**
		G	T			0.011	0.015	0.6794
	Block 3	rs10127190	rs850632	rs179003				
		T	A	T		0.588	0.601	0.7061
		T	G	T		0.200	0.207	0.8161
		T	A	G		0.145	0.135	0.6818
		T	G	G		0.064	0.052	0.4991

*P ≤ 0.05 bolded*.

* *P ≤ 0.05 after 10 000 permutations*.

Haplotype AAC of 4p14 region that included alleles of rs10776482, rs7694115, and rs7660429 was associated in entire vitiligo group (*p* = 0.021, OR 0.71, CI 0.53–0.95) along with stable (*p* = 0.0228, OR 0.59, CI 0.37–0.93), male (*p* = 0.0103, OR 0.54, CI 0.33–0.87), extent < 10% (*p* = 0.019, OR 0.64, CI 0.44–0.93), Köbner (*p* = 0.0427, OR 0.54, CI 0.3–0.99), and late onset (*p* = 0.0149, OR 0.67, CI 0.48–0.93) subgroups. Haplotype AGG of the same block was associated in extent < 10% subgroup (*p* = 0.0031, OR 1.8, CI 1.22–2.67) and this remained significant after correcting for multiple testing (*p* = 0.0331). The other block of this region was formed by rs12233670, rs10005625, rs5743810, and rs6531668. Exception to this was the male vitiligo analysis, where rs6531670 was also added to this block and haplotype TCGGT provided the only association (*p* = 0.021, OR 2.15, CI 1.11–4.16). The other exception in block formation was the female vitiligo analysis where the first block included rs10776482, rs7694115, rs7660429, rs12233670, and rs5743810, and the second block included rs6531668 and rs6531670.

Two haplotype blocks were formed by SNPs of 4q31.3 region. The first block was usually composed of rs1992253, rs4585282, rs7694512, and rs13123230 and the second block of rs1339 and rs2289318. This was not the case in stable, extent < 10% and early onset analyses, where the last SNP of block 1 rs13123230 was transferred to block 2. Additionally, in familial vitiligo analysis rs7694512 of block 1 was also transferred to block 2 and in male vitiligo analysis the first SNP of block 1 was omitted, leaving rs4585282, rs7694512, and rs13123230 in that block. The only haplotype associations found in this region came from block 1, where haplotypes CCG and CCT were associated with stable vitiligo (*p* = 0.034, OR 0.61, CI 0.39–0.97 and *p* = 0.0217, OR 1.7, CI 1.07–2.68, respectively).

The SNPs of 4q35.1 region formed a single haplotype block. It contained all four SNPs of this region, except for male vitiligo analysis, where the last SNP rs1519309 was not included in the block. The strongest associations were uncovered for haplotype AACT in entire vitiligo group (*p* = 7.0E-4, OR 5.84, CI 1.85–18.45) and in active (*p* = 1.0E-4, OR 7.22, CI 2.25–23.23), female (*p* = 0.0018, OR 10.35, CI 1.7–63.1), sporadic (*p* = 0.0051, OR 5.31, CI 1.44–19.67), extent = 10% (*p* = 8.0E-4, OR 7.5, CI 1.87–30.05), spontaneous repigmentation (*p* = 1.0E-4, OR 10.24, CI 2.38–44.02) and late onset (*p* = 0.0018, OR 5.95, CI 1.69–20.96) subgroups. Notably, all these seven associations remained significant after correcting for multiple testing (*p* = 0.0068, *p* = 0.0016, *p* = 0.0139, *p* = 0.0394, *p* = 0.0114, *p* = 0.0023, and *p* = 0.0203, respectively). Haplotype AGCT was associated in familial (*p* = 0.0092, OR 4.3, CI 1.29–14.35), Köbner (*p* = 9.0E-4, OR 6.13, CI 1.82–20.57) and early onset (*p* = 0.0306, OR 3.52, CI 1.04–11.9) subgroups. The result in Köbner group remained significant after correcting for multiple testing (*p* = 0.0141). Haplotypes AAT and AGT were associated with vitiligo in males (*p* = 0.0225, OR 1.75, CI 1.08–2.83 and *p* = 0.0286, OR 0.51, CI 0.27–0.94, respectively).

The two SNPs of 9q33.1 region formed a haplotype block in each of the analyzed groups. Haplotype TA was associated in female (*p* = 0.0401, OR 0.63, CI 0.4–0.98), familial (*p* = 0.0098, OR 0.39, CI 0.19–0.82) and extent ≥ 10% (*p* = 0.0086, OR 0.51, CI 0.3–0.85) subgroups. The last two associations remained significant after correcting for multiple testing (*p* = 0.0198 and *p* = 0.0234, respectively).

Depending on the analyzed group, two or three haplotype blocks were formed by SNPs of Xp22.2 region. Three blocks were present in entire vitiligo group, and active, male, sporadic, extent < 10% and late onset subgroups. Block 1 included rs5935436 and rs179020, block 2 included rs179013 and rs179008, and block 3 included the remaining rs10127190, rs850632 and rs179003. The strongest associations involved block 1 haplotype CA in entire vitiligo (*p* = 5.0E-4, OR 1.75, CI 1.28–2.4), active (*p* = 0.0109, OR 1.59, CI 1.11–2.29), male (*p* = 0.0026, OR 2.23, CI 1.31–3.79), sporadic (*p* = 8.0E-4, OR 1.81, CI 1.28–2.57), extent < 10% (*p* = 0.0094, OR 1.7, CI 1.13–2.54), and late onset (*p* = 2.6E-5, OR 2.1, CI 1.48–2.98) vitiligo groups. From these, the associations in entire vitiligo, sporadic, and late onset vitiligo groups remained significant after correcting for multiple testing (*p* = 0.0244, *p* = 0.04, *p* = 0.0035, respectively). Haplotype CG of the same block was associated in entire vitiligo (*p* = 0.0187, OR 0.7, CI 0.52–0.94), male (*p* = 0.0018, OR 0.45, CI 0.28–0.75), sporadic (*p* = 0.025, OR 0.68, CI 0.49–0.95), extent < 10% (*p* = 0.0246, OR 0.65, CI 0.44–0.95) and late onset (*p* = 0.0028, OR 0.6, CI 0.43–0.84) vitiligo groups. The third haplotype of this block TG was associated with entire vitligo (*p* = 0.0402, OR 0.5, CI 0.25–0.98) as well as sporadic (*p* = 0.035, OR 0.42, CI 0.18–0.97) and late onset vitiligo (*p* = 0.0246, OR 0.38, CI 0.16–0.91). Block 2 haplotypes gave more modest associations when haplotype GA was associated in entire vitiligo (*p* = 0.034, OR 0.72, CI 0.53–0.98) and active vitiligo (*p* = 0.0214, OR 0.66, CI 0.47–0.94) groups and haplotype AT in entire vitiligo (*p* = 0.0275, OR 1.42, CI 1.04–1.95), active (*p* = 0.0272, OR 1.49, CI 1.05–2.12), sporadic (*p* = 0.0449, OR 1.43, CI 1.01–2.02) and late onset (*p* = 0.0408, OR 1.45, CI 1.02–2.06) vitiligo groups. In seven subgroups, namely stable, familial, extent ≥ 10%, Köbner, halo naevi, spontaneous repigmentation and early onset vitiligo the first two haplotype blocks were combined into a single haplotype block containing four SNPs. Haplotype CAAT of this extended block was associated in stable (*p* = 5.0E-4, OR 6.15, CI 1.95–19.46), familial (*p* = 0.0212, OR 4.54, CI 1.13–18.31), and early onset (*p* = 0.0029, OR 5.01, CI 1.56–16.15) subgroups. Haplotypes CGGA and CAGA were both associated in extent ≥ 10% (*p* = 0.034, OR 0.66, CI 0.45–0.97 and *p* = 0.0388, OR 1.55, CI 1.02–2.35, respectively) and halo naevi (*p* = 0.0191, OR 0.42, CI 0.2–0.89 and *p* = 0.012, OR 2.34, CI 1.18–4.62, respectively) subgroups and haplotype TGGA only in extent ≥ 10% subgroup (*p* = 0.0374, OR 0.3, CI 0.09–0.99). One additional version of this first haplotype block was present in female vitiligo analysis. In this instance, SNP rs10127190 was also added to the extended haplotype block and thus it contained five SNPs. Haplotypes TGGAT (*p* = 0.0085, OR 0.26, CI 0.09–0.76) and CAATT (*p* = 0.0302, OR 3.26, CI 1.06–10.01) were both associated with vitiligo in females.

## Discussion

The genotyping procedure in Estonian case-control sample provided data for 29 SNPs. Four allelic and seven haplotype associations were revealed when analysing the entire vitiligo group. In addition, 31 allelic and 48 haplotype associations were present in different subgroups. In terms of genetic location, results with various strength were obtained from all studied regions, except for 1q41 that contains *TLR5* gene.

The most notable SNP in 4p14 region was rs7694115. It was associated in entire vitiligo analysis as well as four subgroup analyses. The given marker is situated in the intron of *TLR10* gene. Two other SNPs of *TLR10* (rs10776482 and rs7660429) and one SNP located in 5′ direction from *TLR10* (rs12233670) were associated in vitiligo subgroups. Of note, rs7660429 and haplotype AGG (rs10776482A, rs7694115G, and rs7660429G) produced a strong association specifically in extent < 10% group, that survived the adjustment for multiple testing. TLR10 protein is expressed by B cells, plasmacytoid dendritic cells and regulatory T cells, but the specific function remains to be clarified since the identity of its ligand has not been established (Hasan et al., 2005; Bell et al., 2007). Previous genetic studies have associated *TLR10* with inflammatory diseases such as asthma (Lazarus et al., 2004), Crohn's disease (Abad et al., 2011; Morgan et al., 2012) and Ménière's disease, that is suspected to have autoimmune properties (Requena et al., 2013). Interestingly, associated SNPs of all these studies fall in the range of here described haplotype block 1 consisting of rs10776482, rs7694115, and rs7660429. It is therefore plausible, that the presently associated SNPs are in linkage disequilibrium with variants that cause changes in TLR10 function and eventually affect disease outcome. There was still another SNP rs6531668 in 4p14 region that was associated in a single subgroup. It is positioned in *TLR6* gene, that has been associated with asthma (Tantisira et al., 2004) and coronary artery disease (Hamann et al., 2013).

One SNP from 4q31.3 region displayed modest associations in vitiligo subgroups. That was rs4585282 located in intron 2 of *TLR2* gene. The respective protein TLR2 recognizes a wide array of antigens and has been suggested to play an instrumental role in the development of self-reactive diseases (Borrello et al., 2011). In the skin, TLR2 is expressed in antigen-presenting Langerhans cells, as well as keratinocytes and melanocytes (Yu et al., 2009; Hari et al., 2010). Polymorphisms in this gene have been associated with autoimmune diseases, namely type 1 diabetes, allergic asthma (Bjornvold et al., 2009), severe ulcerative colitis (Pierik et al., 2006) and also atopic dermatitis, that is proposed to hold autoimmune qualities (Ahmad-Nejad et al., 2004; Cipriani et al., 2014). More intriguingly, the Arg753Gln (rs5743708) polymorphism was associated with vitiligo in Turkish patients (Karaca et al., 2013). It is located 14 kb from the currently associated rs4585282 and is a missense mutation causing arginine to glutamine substitution in TLR2 peptide. The haplotype block 1 that contains rs4585282 did not reach over the position of Arg753Gln and the possible relationship between these two polymorphisms together with effects on TLR2 activity remain to be determined.

The four SNPs of 4q35.1 were in the region of *TLR3* gene. SNP rs6552950 from *TLR3* intron produced the only allelic association with vitiligo among males. However, several strong haplotype associations appeared in entire vitiligo and subgroup analyses, that remained statistically significant after correcting for multiple testing. The TLR3 receptor recognizes double-stranded (ds)RNA during viral infection and initiates the production of type I interferons in response. It has been shown that TLR3 is also expressed in melanocytes and stimulation with dsRNA leads to apoptosis (Yu et al., 2011). To date, polymorphisms of *TLR3* have been associated with several chronic inflammatory diseases e.g., rheumatoid arthritis (Laska et al., 2014a), systemic lupus erythematosus (SLE) (Laska et al., 2014b) and osteoarthritis (Yang et al., 2013), the first two of which are autoimmune diseases. Interestingly, the associated polymorphisms in these reports (rs3775296, rs5743312, rs3775291, and rs3775290) fall in the range of a haplotype block formed by the SNPs genotyped in this study. Therefore, it is possible that these associations signal the existence of certain causal polymorphisms within the block, such as the non-synonymous rs3775291, that affect vitiligo pathology.

A single SNP rs5030728 from 9q33.1 region was associated in familial, extent ≥ 10% and female subgroups. Likewise, haplotype TA consisting of rs10759932T and rs5030728A was associated in the same groups. Notably, the results from familial and extent ≥ 10% groups remained significant when corrections for multiple testing were applied. The former SNP is located near the 5′ end of *TLR4* gene and the latter is an intronic SNP of *TLR4*. This receptor is known for recognizing lipopolysaccharide from gram-negative bacteria, which induces the production of proinflammatory cytokines and Type I interferons (Lu et al., 2008). As a contributor to TLR2/TLR4 pathway, it has been attributed a pivotal function in the pathogenesis of autoimmune diseases (Liu et al., 2014). Similarly, to TLR2 and TLR3, it has been detected in Langerhans cells, keratinocytes and melanocytes (Hari et al., 2010). Polymorphisms of *TLR4* have been associated with Crohn's disease, ulcerative colitis (Shen et al., 2010b), asthma (Zhang et al., 2011) and atherosclerosis (Kiechl et al., 2002). Remarkably, the Asp299Gly (rs4986790) polymorphism was also associated with vitiligo among Turkish patients (Karaca et al., 2013). It is positioned 1 kb from rs5030728 and while not situated within here described haplotype block, it may still be in linkage disequilibrium with these SNPs. Hence, it is possible that the current associations with non-coding SNPs reflect the effect of missense Asp299Gly on TLR2 activity. Conversely, they may have an independent impact and rs5030728 has been suggested to play a role in metabolic syndrome (Cuda et al., 2011) and inflammatory bowel disease treatment response (Bank et al., 2014). The other contributor to TA haplotype described above, rs10759932, has been associated with prostate cancer (Cheng et al., 2007), chorionic plate inflammation (Simhan et al., 2008) and atopy (Penders et al., 2010).

The final studied region was in Xp22.2 that contained *TLR7* and *TLR8* genes. The strongest associations were obtained with SNP rs179020 located in intron 2 of *TLR7*. Notably, this SNP remained significant after correcting for multiple testing in entire vitiligo and three subgroup analyses: male, sporadic, and late onset vitiligo. Furthermore, allele rs5935436C together with rs179020A formed a haplotype CA, that withstood the correction in all these groups except vitiligo males. Three other SNPs from *TLR7* region—rs5935436, rs179013, and rs179008—produced weaker allelic associations in entire vitiligo and subgroup analyses. TLR7 is an intracellular receptor located in endosomes, that binds single-stranded RNA and induces the production of inflammatoty cytokines (Diebold et al., 2004). In addition to immune cells, its expression has been demonstrated in melanocytes (Kang et al., 2009). It is intriguing to note, that topical administration of TLR7 agonist imiquimod has been shown to be associated with vitiligo-like hypopigmentation (Brown et al., 2005). Considering that TLR7 activation may lead to apoptosis (Meyer et al., 2003), this effect could be due to TLR7 induced loss of melanocytes. Concurrently, the role of TLR7 in other autoimmune diseases has been widely reported, especially in the case of SLE (Deane et al., 2007; O'Neill et al., 2009). The proposed mechanism involves activation of autoreactive B cells through TLR7 signaling, while TLR9 acts as a restrictor of this process and the role of TLR8 is yet to be clarified (Soni et al., 2014). On a genetic level, *TLR7* SNPs have been associated with Graves' disease (Xiao et al., 2015) and SLE (Shen et al., 2010a; Enevold et al., 2014). Among the four SNPs associated in this study, rs179008 has recently been associated with asthma (Møller-Larsen et al., 2008), multiple sclerosis (Enevold et al., 2010), and SLE (dos Santos et al., 2012). It is a non-synonymous mutation, where A to T substitution causes glycine to leucine change in TLR7 peptide (Gln11Leu). Currently, T was the associated allele, whereas previously both A and T were shown to confer disease susceptibility (Møller-Larsen et al., 2008; dos Santos et al., 2012). In silico analyses by Møller-Larsen and colleagues suggested, that changing Gln11 to Leu shortens the N-region and extends the H-region of TLR7 signal peptide, thus potentially affecting post-translational processing (Møller-Larsen et al., 2008). Taken together, these genetic associations of four *TLR7* SNPs could indicate alterations in TLR7 functioning, but the precise mechanisms remain to be determined in future studies.

In conclusion, the present study revealed a number of genetic associations between TLR gene SNPs and vitiligo susceptibility. The main findings emerged with *TLR7* SNP 179020 and *TLR3* haplotype AACT. SNPs of *TLR10* and *TLR4* were associated in specific subgroups, indicating their possible role in certain subphenotypes. However, the interpretation of these results should account for the small sample used, while the functional effects of studied SNPs remain to be elucidated in future experiments.

### Conflict of interest statement

The authors declare that the research was conducted in the absence of any commercial or financial relationships that could be construed as a potential conflict of interest.
